# Disordered eating and self-harm as risk factors for poorer mental health during the COVID-19 pandemic: A population-based cohort study

**DOI:** 10.1101/2021.04.30.21256377

**Published:** 2021-05-04

**Authors:** Naomi Warne, Jon Heron, Becky Mars, Alex S. F. Kwong, Francesca Solmi, Rebecca Pearson, Paul Moran, Helen Bould

**Affiliations:** 1 Centre for Academic Mental Health, Population Health Sciences, Bristol Medical School, University of Bristol, UK; 2 Population Health Sciences, Bristol Medical School, University of Bristol, UK; 3 MRC Integrative Epidemiology Unit, Bristol Medical School, University of Bristol, UK; 4 Division of Psychiatry, Centre for Clinical Brain Sciences, University of Edinburgh, UK; 5 UCL Division of Psychiatry, UK; 6 Gloucestershire Health and Care NHS Foundation Trust, Gloucester, UK

**Keywords:** ALSPAC, COVID-19, disordered eating, self-harm, mental health, pandemic, lockdown

## Abstract

**Background:**

Young adults and especially those with pre-existing mental health conditions, such as disordered eating and self-harm, appear to be at greater risk of developing metal health problems during the COVID-19 pandemic. However, it is unclear whether this increased risk is affected by any changes in lockdown restrictions, and whether any lifestyle changes could moderate this increased risk.

**Methods:**

In a longitudinal UK population-based cohort (The Avon Longitudinal Study of Parents and Children, ALSPAC) we assessed the relationship between pre-pandemic measures of disordered eating and self-harm and mental health during the COVID-19 pandemic in 2,657 young adults. Regression models examined the relationship between self-reported disordered eating, self-harm, and both disordered eating and self-harm at age 25 years and depressive symptoms, anxiety symptoms and mental wellbeing during a period of eased restrictions in the COVID-19 pandemic (May-July 2020) when participants were aged 27–29 years. Analyses were adjusted for sex, questionnaire completion date, pre-pandemic socioeconomic disadvantage and pre-pandemic mental health and wellbeing. We also examined whether lifestyle changes (sleep, exercise, alcohol, visiting green space, eating, talking with family/friends, hobbies, relaxation) in the initial UK lockdown (April-May 2020) moderated these associations.

**Results:**

Pre-existing disordered eating, self-harm and comorbid disordered eating and self-harm were all associated with the reporting of a higher frequency of depressive symptoms and anxiety symptoms, and poorer mental wellbeing during the pandemic compared to individuals without disordered eating and self-harm. Associations remained when adjusting for pre-pandemic mental health measures. There was little evidence that interactions between disordered eating and self-harm exposures and lifestyle change moderators affected pandemic mental health and wellbeing.

**Conclusions:**

Young adults with pre-pandemic disordered eating, self-harm and comorbid disordered eating and self-harm are at increased risk for developing symptoms of depression, anxiety and poor mental wellbeing during the COVID-19 pandemic, even when accounting for pre-pandemic mental health. Lifestyle changes during the pandemic do not appear to alter this risk. A greater focus on rapid and responsive service provision is essential to reduce the impact of the pandemic on the mental health of these already vulnerable individuals.

## Plain English summary

The aim of this project was to explore the mental health of young adults with disordered eating behaviours (such as fasting, vomiting/taking laxatives, binge-eating and excessive exercise) and self-harm during the COVID-19 pandemic. We analysed data from an established study that has followed children from birth (in 1991 and 1992) up to present day, including during the pandemic when participants were 28 years old. We looked at the relationship between disordered eating and/or self-harm behaviours from *before* the pandemic and mental health problems (symptoms of depression and anxiety) and mental wellbeing *during* the pandemic. We also explored whether there were any lifestyle changes (such as changes in sleep, exercise, visiting green space) that might be linked to better mental health and wellbeing in young adults with disordered eating and self-harm. We found that young adults with prior disordered eating and/or self-harm had more symptoms of depression and anxiety, and worse mental wellbeing than individuals without prior disordered eating or self-harm. However, lifestyle changes did not appear to affect mental health and wellbeing in these young adults. Our findings suggest that people with a history of disordered eating and/or self-harm are at high risk for developing mental health problems during the pandemic, and they will need help from mental health services.

## Background

The coronavirus disease 2019 (COVID-19) pandemic has had an immense impact on people’s lives worldwide. On 23^rd^ March 2020, a national lockdown was announced as a UK public health strategy instructing the public to stay at home except for certain limited purposes. During this time, people could only leave their homes once a day for one hour for exercise or shopping for essential goods (e.g. food, medicine). Non-essential businesses were closed, schools were closed for the majority of students (exceptions were for vulnerable children and children of keyworkers), and people were urged to work from home where possible. Restrictions were gradually eased, allowing for unlimited exercise outside (13^th^ May 2020), groups of up to six people to meet outside with social distancing (1^st^ June 2020), year groups returning to schools (1^st^ June 2020), and non-essential shops re-opening (15^th^ June 2020). The pandemic and associated restrictions radically changed people’s lives and there is evidence that mental health in the UK population was worse during lockdown than before the pandemic (1–4). Furthermore, young adults and individuals with prior mental health problems were at increased risk of common mental health problems (depressive symptoms and anxiety symptoms) during the UK lockdown (1).

Young adults with pre-existing common mental health problems are likely to be at particularly high risk of experiencing poor mental health during the pandemic. Eating disorder behaviours and self-harm are common in young adults (5) and are associated with increased risk of mortality and psychiatric comorbidity (6–9). They commonly co-occur in clinical (10) and general population (5) samples, and this comorbidity is a great clinical concern as it increases the risk of poorer overall mental health (11–13) and risk for suicide (14), compared with when the behaviours occur in isolation. Furthermore, there is growing concern that individuals with a history of eating disorders and/or self-harm may have been particularly affected by the pandemic and the associated restrictions. In addition to the broader risk factors that can affect many people (for instance social isolation, stressful life events) individuals with eating disorders may experience more specific risk factors that can lead to increased distress such as changes in access to food, exercise limitations, media messaging and restricted healthcare access (15,16). Individuals with disordered eating are also likely to experience some of these more specific risk factors. Similarly, the pandemic has resulted in a number of exacerbating factors for self-harm (17) and the reductions of clinical services presentations for self-harm may reflect reduced help-seeking in this vulnerable group (18,19). Given these additional risk factors, individuals with eating disorders and self-harm may be a key ‘at risk’ group for poorer mental health (such as depression and anxiety) during the pandemic. Prior research has found that disordered eating and self-harm are risk factors for increased depressive and anxiety symptoms in young adults during the first UK lockdown (1). However, it is unclear whether this increased risk persists when lockdown restrictions have eased, and whether lifestyle factors could play a role in this risk.

To date, there has been limited published research on COVID-19-related common mental health outcomes in individuals with eating disorders and self-harm. One cross-sectional Australian study found that individuals self-reporting an eating disorder had higher levels of depression, anxiety and stress than individuals not reporting an eating disorder in April 2020 (20). Cross-sectional studies have found the majority of participants with eating disorders have also self-reported a worsening of depressive symptoms (21) and anxiety symptoms (22,23) due to the pandemic, however a longitudinal study found no change in general psychopathology (including depression and anxiety) pre- to post-lockdown in eating disorder patients (24). There is very limited research for pandemic common mental health problems in those with a history of self-harm but there is emerging evidence that individuals with a previous history of self-harm with suicidal intent (suicide attempt) were more likely to have depression in a Greek lockdown (April-May 2020) than those without a history of suicide attempt (25).

Although informative, conclusions from these studies are limited by the self-selected participants, the small sample sizes (typically under ~200 people) and the retrospective reporting of changes in mental health. Furthermore, many studies focus on clinical samples, whereas only a minority of individuals with disordered eating and self-harm seek help (6,26). Research using longitudinal population-based samples with pre-pandemic measures of mental health can provide a more accurate representation of pandemic-related effects on mental health in these groups. Such samples include a more representative sample of participants than those found from convenience sampling and include participants with disordered eating or self-harm who may be missed by clinical services.

In this study, we used a UK general population cohort 1) to investigate whether prior disordered eating and self-harm were risk factors for higher levels of depression and anxiety and lower levels of mental wellbeing during a period of eased restrictions in the COVID-19 pandemic; and 2) to assess whether lifestyle changes can identify individuals with prior disordered eating and self-harm who may have better mental health in the pandemic.

## Methods

### Sample

We analysed data on young adults from a population-based birth cohort: The Avon Longitudinal Study of Parents and Children (ALSPAC) (27–29). Pregnant women living in the Avon area of Bristol (UK) with an expected delivery date between 1^st^ April 1991 and 31^st^ December 1992 were invited to take part in the study. 14,541 pregnant women were enrolled in ALSPAC and had completed at least one assessment (questionnaire or in person clinic) by 19^th^ July 1999. This resulted in 14,062 live births, and 13,988 children who were alive at 1 year of age. We used data on offspring from this core sample only. Ethical approval for the study was obtained from the ALSPAC Ethics and Law Committee and the Local Research Ethics Committees.

**Figure 1.**
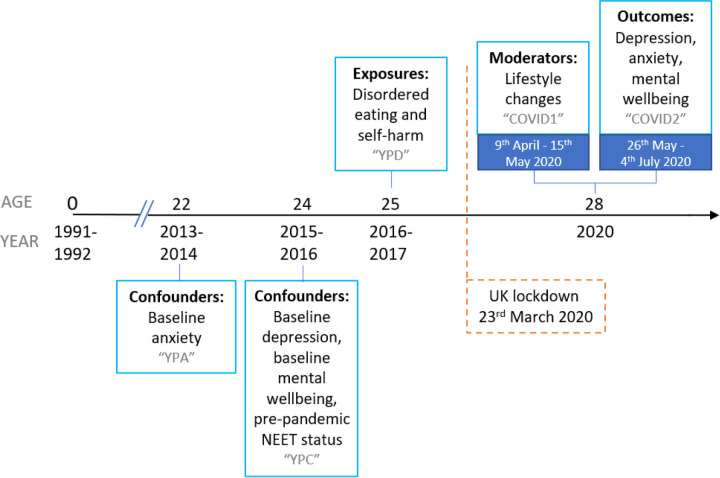
Timeline of ALSPAC assessments NEET = Not in education, employment or training. Grey text indicates the name of the ALSPAC questionnaire.

Data were collected at multiple time points and from multiple informants via regular questionnaires and face-to-face assessments at research clinics. Some of these data were collected and managed using REDCap electronic data capture tools (30,31). Please note that the study website contains details of all the data that is available through a fully searchable data dictionary and variable search tool: http://www.bristol.ac.uk/alspac/researchers/our-data/. In this study, we focused on exposures of disordered eating and self-harm behaviours reported by participants *before* the pandemic at age 25 years, and additional data on other health characteristics, collated from two online questionnaires sent following UK lockdown (which started on 23rd March 2020). Mental health and wellbeing outcomes were self-reported during a period of eased restrictions (26th May - 4th July 2020) in the COVID-19 pandemic when participants were age 28 years (32). We also looked at self-reported lifestyle changes during the initial UK lockdown (9th April - 15th May 2020) (33) as potential moderators of this relationship (see timeline of assessments in Figure 1).

We conducted our primary analyses on an imputed sample of 2657 individuals (1891 (71.17%) females, 766 (28.83%) males) who completed questions on lifestyle changes during the pandemic (see Supplementary Figure 1 for flowchart of attrition). Responders to this survey were more likely than non-responders to be female, white, in education, employment or training prior to the pandemic, and have a higher maternal education and have parents who owned a home around birth (see Supplementary Table 1).

### Exposures

Disordered eating and self-harm were self-reported via questionnaire at age 25 (“YPD” questionnaire). Age at completion ranged from 23.8 years to 26.3 years with a mean age of 24.8 (SD 0.5) years.

#### Disordered eating

Disordered eating was measured using the Youth Risk Behaviour Surveillance System questionnaire (34). We used questions about behaviours in the last year to lose weight or avoid gaining weight: 1) fasting for at least a day; 2) purging (vomiting or taking laxatives/other medications); 3) excessive exercise (exercise that frequently interfered with daily routine/work, or frequently exercising even when sick/injured); as well as 4) binge-eating with a sense of loss of control. Our primary exposure of interest was *any disordered eating*, a composite measure derived for any report of fasting, purging, excessive exercise or binge-eating at any frequency. Secondary exposures of interest were each of the individual disordered eating behaviours (fasting, purging, binge-eating, excessive exercise) at any frequency, and a composite measure of any of these behaviours at least once a week in line with diagnostic criteria (35): *DSM-5 disordered eating*. Questions, possible responses and variable derivation are presented in Supplementary Table 2.

#### Self-harm

We assessed self-harm using questions adapted from the Child and Adolescent Self-Harm in Europe study (36). We used self-harm behaviour in the last year, in order to be comparable with our measures of disordered eating. Participants were asked a series of questions about the presence and frequency of self-harm behaviours (see Supplementary Table 2). Our primary exposure of interest was *any self-harm,* regardless of suicidal intent, in the past year. This was derived from questions asking whether participants had hurt themselves on purpose in any way and how many times they did this in the last year. Secondary exposures of interest were *self-harm without suicidal intent* and *self-harm with suicidal intent (suicidal attempt)*. Self-harm without suicidal intent was reported if, when asked the question “when was the last time you hurt yourself on purpose, without intending to kill yourself?”, they responded with “in the last week”, or “more than a week ago but in the last year”. Similarly, participants with those responses to the question “when was the last time you hurt yourself on purpose and you seriously wanted to kill yourself?” were recorded as having *self-harm with suicidal intent*. Participants could therefore report both self-harm *with* suicidal intent and self-harm *without* suicidal intent in the last year.

#### Comorbid disordered eating and self-harm

Individuals who reported *any disordered eating* (at any frequency) and *any self-harm* were coded as having comorbid disordered eating and self-harm behaviours.

### Outcomes

Outcomes were taken from the second COVID-19-related questionnaire (“COVID2” questionnaire) which was sent to participants during a period of eased restrictions between 26th May and 4th July 2020. Participants were aged 27–29 years at completion (mean (SD) = 28.2 (0.5) years).

#### Depressive symptoms during the COVID-19 pandemic

Depressive symptoms were measured using the short version of the Mood and Feelings Questionnaire (sMFQ) (37). Participants reported whether 13 depressive symptom statements were ‘not true’ (0), ‘sometimes true’ (1), and ‘true’ (2) for the previous 2 weeks. Scores were summed (possible range 0–26) with higher scores indicating more depressive symptoms.

#### Anxiety symptoms during the COVID-19 pandemic

Anxiety symptoms were measured on the Generalised Anxiety Disorder 7-item questionnaire (GAD-7) (38). Participants reported whether they had been bothered by each of the 7 anxiety statements in the past 2 weeks on the response scale ‘not at all’ (0), ‘several days’ (1), ‘more than half the days’ (2), and ‘nearly every day’ (3). These responses were summed for a total score with possible range 0–21, with higher scores indicating higher levels of anxiety.

#### Mental wellbeing during the COVID-19 pandemic

Mental wellbeing was measured on the Warwick-Edinburgh Mental Well-Being Scale (WEMWBS) (39). This consisted of 14 wellbeing statements that participants rated over the past 2 weeks on a scale of ‘none of the time’ (1), ‘rarely’ (2), ‘some of the time’ (3), ‘often’ (4), and ‘all of the time’ (5). Summed scores produced a possible range of 14–70 with lower scores indicating poorer mental wellbeing.

### Moderating factors

Participants reported on a number of lifestyle changes that occurred after the first UK lockdown was announced (23^rd^ March 2020) in the first online COVID-19-related questionnaire (“COVID1” questionnaire) (10). This questionnaire was sent during lockdown between 9th April and 15th May 2020 (mean (SD) age = 28.1 (0.5) years). Questions about lifestyle changes included changes in the amount of sleep, exercise, alcohol drunk, visiting green space, practising relaxation/mindfulness/mediation, eating, as well as changes in time spent talking to family/friends outside their home and time spent doing hobbies/things they enjoy. Participants reported whether the amount they did each activity had “decreased”, “stayed the same”, “increased” or was “not applicable” since the first UK lockdown (23^rd^ March 2020). For our analyses we combined “not applicable” with “stayed the same” as we made the assumption that the majority of people responding “not applicable” likely maintained an absence of the activity.

### Confounders and descriptive variables

Based on their plausible associations with exposures, outcomes and moderating factors, we adjusted for hypothesised confounders of sex, completion date of the COVID1 questionnaire, pre-pandemic socioeconomic status and baseline mental health symptoms. Participation in education, employment or training activities was used as the pre-pandemic indicator of socioeconomic status, with those not in education, employment, or training (NEET) designated a being in the socioeconomic disadvantage category. Participants reported on their education and employment status, prior to the pandemic (2015–2016) at age 24 years. For baseline mental health symptoms, we used the most recent reports on the same questionnaires preceding the disordered eating and self-harm exposures (age 25). For depressive symptoms (sMFQ) and mental wellbeing (WEMWBS) this was at age 24 years and for anxiety symptoms (GAD-7) this was at age 22 years. Timings of all exposures, outcomes and confounders are presented in Figure 1.

We also described the sample’s sociodemographic characteristics, and pandemic-related experiences, the latter were measured on the COVID2 questionnaire. Participants were categorised as *living alone* if they responded “no I live on my own” to the question “Do you live with anyone?”. Participants were defined as a *keyworker* if they responded “yes” to the question: “Are you a keyworker, or has your work been classified as critical to the COVID-19 response?”. Participants were considered to be *furloughed during the pandemic* if they responded “yes” to the question: “Which of these would you say best describes your current situation now?: Employed but on paid leave (including furlough)”. For pandemic financial situation, participants were asked: “Overall, how do you feel your current financial situation compares to how it was before the COVID-19 pandemic?” with possible responses of “I’m much worse off”, “I’m a little worse off”, “I’m about the same”, “I’m a little better off”, “I’m much better off”. Individuals responding “I’m much worse off” and “I’m a little worse off” were coded as having *financial problems during the pandemic*.

### Analysis

All analyses were performed in Stata version 16 (40). First, we described the samples in terms of sex, ethnicity, sociodemographic factors, pandemic-related experiences, and key exposure and outcome variables. Secondly, we used linear regression models to explore the relationship between disordered eating and self-harm exposures and the three mental health and wellbeing outcomes (depressive symptoms, anxiety symptoms, mental wellbeing) during a period of eased restrictions in the COVID-19 pandemic. We focused on our primary exposures of *any disordered eating*, *any self-harm* and *comorbid disordered eating and self-harm*. Analyses were repeated with secondary exposures of specific types of disordered eating (fasting, purging, excessive exercise, binge eating, DSM-5 disordered eating) and self-harm with and without suicidal intent. We conducted these analyses unadjusted for confounders (Model A) and with sequential adjustment to assess the effects of confounders. We progressively adjusted for: sex, COVID1 questionnaire completion date, pre-pandemic NEET status (Model B); and corresponding baseline mental health symptoms/wellbeing (Model C). We examined whether the association between exposure and outcome varied depending on changes in lifestyle factors by testing whether there was evidence of an interaction between lifestyle change and disordered eating status, and lifestyle change and self-harm status on pandemic mental health and wellbeing. To aid in interpretation of interaction results we also examined the association between disordered eating and self-harm exposures and lifestyle change moderators, as well as associations between lifestyle change factors and pandemic-related mental health outcomes.

#### Missing data

Primary analyses were conducted on an imputed dataset of 2657 individuals with complete data on lifestyle change moderators measured on the COVID1 questionnaire. Missing data on primary exposures, outcomes, and confounders were imputed (see Supplementary Table 3 for amount of missing data). Secondary exposures were not imputed, and we did not impute data on lifestyle changes in COVID1 questionnaire or pandemic-related descriptive factors as these data were unique and unlikely to be explained by auxiliary data in ALSPAC. Data were imputed using the multivariate imputation by chained equations (MICE) approach (41) under the missing at random (MAR) assumption. In addition to variables used in the main analysis, we incorporated auxiliary variables related to the missing data mechanism. Auxiliary variables were disordered eating at 16 and 18 years, self-harm at 18 and 24 years, Body Mass Index at 18 and 24 years, and mental health and wellbeing measures from the COVID1 questionnaire. A number of sociodemographic variables collected near birth including maternal age, parity, maternal education, maternal social class, paternal social class, home ownership status and birthweight were also used. One hundred datasets were imputed (a decision informed by studying the Monte Carlo errors for the estimated parameters). Separate multiple imputation models were performed: one model for the main regression models and multiple imputation models including all interactions (42) to allow for interactions between each exposure and each lifestyle change variable. We compared estimates from imputed analysis to analysis of observed data with complete information on disordered eating, self-harm, mental health measures during the pandemic, lifestyle change moderators, and confounders (see flowchart of attrition, Supplementary Figure 1).

## Results

### Descriptive results

Descriptive information for the imputed and observed samples is presented in Table 1. As can be seen, the sample was predominantly white and in education, employment or training before the pandemic. During the pandemic (observed sample), 7.20% were living alone, approximately two in five (39.66%) participants were keyworkers, one in eight (12.31%) had been furloughed, and a quarter (24.61%) reported financial problems. At age 25 (imputed sample), 32.04% of the sample reported some form of disordered eating in the past year, 8.97% reported self-harm in the past year, and 5.53% reported comorbid disordered eating and self-harm in the past year. The most common specific disordered eating behaviour (observed sample) was binge-eating (20.46%), whereas the most common type of self-harm reported was self-harm without suicidal intent (6.19%).

### Associations between disordered eating/self-harm and mental health outcomes during the pandemic

Regression models testing the associations between disordered eating and self-harm exposures with mental health and wellbeing outcomes are presented in Table 2. There was evidence that a history of any disordered eating, any self-harm, and comorbid disordered eating and self-harm were associated with higher depressive and anxiety symptoms, and lower mental wellbeing during the pandemic. These associations were present in unadjusted models, and remained unchanged when adjusting for sex, COVID questionnaire completion date and pre-pandemic NEET status. There was still strong evidence of these associations after further adjustment for baseline mental health and wellbeing although the magnitude of the associations was attenuated. Results using imputed data were consistent with complete case observed data (see Supplementary Table 4).

Broadly similar patterns were detected for analyses with secondary exposures (see Supplementary Table 5). Subtypes of disordered eating (fasting, purging, binge-eating, any DSM-5 disordered eating) and self-harm (with and without suicidal intent) were associated with greater depressive symptoms, greater anxiety symptoms and lower mental wellbeing during the pandemic in unadjusted and in adjusted models. However, excessive exercise was not associated with pandemic depressive symptoms and mental wellbeing in fully adjusted models (Supplementary Table 5). In addition, self-harm without suicidal intent was no longer associated with mental wellbeing in the pandemic when accounting for baseline mental wellbeing (B = −1.28 (95% CI −2.93, 0.37), p = 0.127).

### Moderation of lifestyle changes

**Figure 2.**
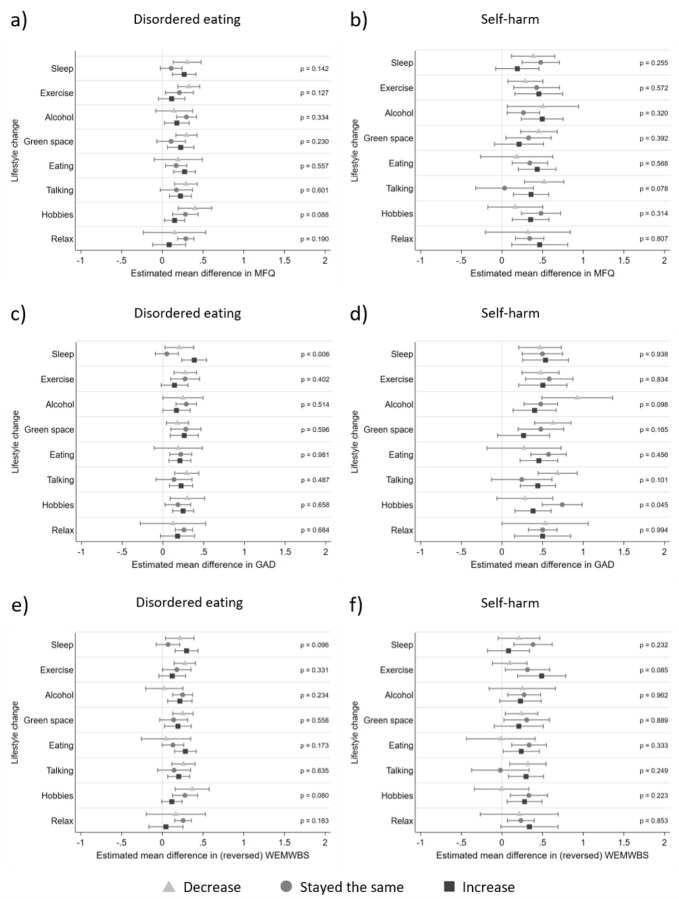
Adjusted associations between disordered eating/self-harm and mental health and wellbeing stratified by moderators (lifestyle changes) Panel a) Association between disordered eating and depressive symptoms, stratified by moderators; Panel b) Association between self-harm and depressive symptoms, stratified by moderators; Panel c) association between disordered eating and anxiety symptoms, stratified by moderators; Panel d) association between self-harm and anxiety symptoms, stratified by moderators; Panel e) association between disordered eating and mental wellbeing, stratified by moderators; Panel f) association between self-harm and mental wellbeing, stratified by moderators. All associations were adjusted for sex, date of completion for COVID questionnaires, pre-pandemic NEET (not in education, employment or training) status and baseline symptoms. Results using imputed data (n=2657). P values displayed are for the interactions between exposure and moderator on mental health and wellbeing outcomes. MFQ = Mood and Feelings Questionnaire for depressive symptoms GAD = Generalised Anxiety Disorder 7-item questionnaire for anxiety symptoms WEMWBS = Warwick-Edinburgh Mental Well-Being Scale for mental wellbeing

Interaction results are presented in Figure 2 alongside associations between disordered eating/self-harm exposures and pandemic mental health and wellbeing outcomes stratified by levels of lifestyle change moderators to aid interpretation (see Supplementary Table 6 for frequencies of each lifestyle change). As can be seen, irrespective of lifestyle change and outcome, disordered eating and self-harm are all associated with worse mental health outcomes, but there was little evidence of interaction effects between lifestyle changes during the pandemic and prior disordered eating (Figure 2a, 2c and 2e) and prior self-harm (Figure 2b, 2d and 2f). There was evidence for one interaction between disordered eating and sleep on anxiety symptoms (Figure 2c, F = 5.07, p = 0.006), in that keeping the same level of sleep was associated with lower levels of anxiety than changes (decreases and increases) in sleep for those with disordered eating. This pattern of results was also seen for depressive symptom and mental wellbeing outcomes but there was little evidence for interaction effects (depressive symptoms Figure 2a, F = 1.96, p =.142; mental wellbeing Figure 2e, F = 2.35, p = 0.096). There was an interaction between self-harm and hobbies for anxiety symptoms (Figure 2d, F = 3.10, p = 0.045), whereby changes (increases and decreases) in time spent on hobbies was associated with fewer anxiety symptoms for those with a history of self-harm.

There was evidence for some associations between disordered eating and self-harm exposures and lifestyle changes (Supplementary Table 7), and changes (decreases and increases) in lifestyle were generally associated with poorer subsequent mental health and wellbeing compared to maintaining the same level of each lifestyle factor (Supplementary Table 8).

## Discussion

In this UK population-based cohort, young adults with a previous history of either disordered eating, self-harm, or comorbid disordered eating and self-harm, were at increased risk for poor mental health and wellbeing during the COVID-19 pandemic. Specifically, disordered eating, self-harm and comorbid disordered eating and self-harm were associated with higher depressive symptoms, higher anxiety symptoms and poorer mental wellbeing during a period of easing restrictions. Associations attenuated somewhat with the addition of prior mental health and wellbeing confounders, but there was still evidence of associations between disordered eating and self-harm exposures and pandemic mental health outcomes independent of pre-pandemic levels of mental health and wellbeing. Furthermore, there was limited evidence that any changes in lifestyle (sleep, exercise, drinking alcohol, visiting green space, eating, talking with family/friends outside the home, hobbies, relaxation techniques) during lockdown restrictions moderated these relationships. These findings suggest that young adults with disordered eating and self-harm may need additional help to prevent mental health problems from developing during the pandemic or for rapid access to treatment.

Previous research in this cohort has found individuals with a history of disordered eating (at DSM-5 frequency (35)) and self-harm are associated with higher levels of depressive and anxiety symptoms during the initial UK lockdown (1). The current study extends these findings to a period of eased restrictions and suggests that individuals with disordered eating and self-harm may be at enduring risk for common mental health problems throughout the COVID-19 pandemic. Clearly policymakers and healthcare professionals should be aware that individuals with disordered eating and self-harm are at greater risk of experiencing worse mental health outcomes during this period of pandemic. Importantly, our results find that the association between a history of disordered eating behaviours and poorer mental health during the pandemic is not limited to more severe DSM-5 level frequencies but also extends to any level of disordered eating. Therefore, greater funding for mental health services should be prioritised to provide help to individuals across a range of severities, as early as possible to prevent negative effects of enduring mental health problems.

We found little evidence that lifestyle changes could moderate these risks. There was weak evidence for interactions with a number of different lifestyle changes but given the numerous of moderation tests conducted we are cautious about interpreting this weak evidence. However, we found evidence for an interaction between disordered eating and sleep on anxiety symptoms, whereby maintaining pre-pandemic levels of sleep was associated with fewer anxiety symptoms than increases or decreases in sleep for those with a history of disordered eating. This is difficult to interpret given lifestyle changes in the pandemic (in this case sleep) are unlikely to be independent of exposures and outcomes (see Supplementary Tables 7 and 8) and may be affected by concurrent or recent changes in mental health, employment, and childcare responsibilities. In general, changes in the lifestyle factors assessed were associated with worse mental health and wellbeing regardless of prior disordered eating or self-harm (Supplementary Table 8). Future work focusing on factors that promote resilience in these at risk groups, who are already at elevated risk of mortality (7,9), is warranted. It is not clear what would be useful for the mental health of young adults with prior disordered eating and self-harm during the pandemic, but it is likely that access to mental health services will be important. This is difficult in the context of the pandemic when service use has reduced (43) and, therefore, additional funding that enables greater service provision, adapted for pandemic-related restrictions, is vital.

The strengths of this study include the large sample size, the use of longitudinal data to assess the effect of the pandemic on mental health by accounting for pre-pandemic mental health and use of population-based participants with impairing disordered eating and self-harm who would normally be omitted from clinical samples. Nevertheless, the study should be viewed in light of its limitations. Firstly, we were unable to explore disordered eating and self-harm during the pandemic as there was no data available for this. Secondly, we used imputed datasets under the assumption that data is missing at random, which if not true, could mean the results are biased. Furthermore, we only imputed up to those with complete lifestyle change data on the COVID1 questionnaire and this may limit generalisability of findings as this sample was more likely to be female, white and have less socioeconomic disadvantage than those who did not respond to the survey. Thirdly, we did not have sufficient numbers of people with different ethnic backgrounds to assess the effect of ethnicity on the relationships between disordered eating, self-harm and pandemic mental health. Fourthly, interpretation of our moderation results is difficult given lifestyle change moderators are not independent of the exposures and outcomes, and may be associated with additional factors such as employment change. Consequently, we were unable to make inferences about the direction of effect, and this required further exploration. Finally, we did not correct for multiple comparisons due to the exploratory nature of analyses and the lack of independence of many of the measures. These results are therefore in need of replication in independent samples.

## Conclusions

Individuals with prior disordered eating and self-harm were at increased risk of developing common mental health problems during the COVID-19 pandemic. There was little evidence to support lifestyle changes moderating this risk. Further work is needed to identify factors that might increase resilience among individuals with disordered eating and self-harm during the pandemic in order to prevent them from developing common mental health problems. Additional funding for mental health services will likely be important to provide rapid treatment for these at risk young adults.

## Supplementary Material

Supplement 1

## Figures and Tables

**Table 1.** Descriptive information on imputed and observed samples

	Imputed sample	Observed sample		

		Total sample	Any disordered eating	Any self-harm
	Sample mean (SE) or % (SE)	Sample mean (SE) or % (n)	Sample mean (SE) or % (n)	Sample mean (SE) or % (n)
Sex (female)		71.17% (1891/2657)	84.52% (557/659)	85.41% (158/185)
Ethnicity (non-white)	3.50% (0.37)	3.42% (87/2542)	3.06% (19/621)	** **
Not in education, employment or training before the pandemic	5.36% (0.53)	5.04% (100/1986)	5.83% (32/549)	5.92% (9/152)
Living alone in the pandemic		7.20% (139/1931)	9.18% (46/501)	9.46% (14/148)
Keyworker in the pandemic		39.66% (742/1871)	41.02% (201/490)	33.79% (49/145)
Furloughed during the pandemic		12.31% (327/2657)	12.29% (81/659)	15.14% (28/185)
Financial problems during the pandemic		24.61% (474/1926)	27.15% (136/501)	25.68% (38/148)
Any disordered eating	32.04% (1.01)	31.44% (659/2096)		
Fasting		10.78% (228/2115)		
Purging		9.04% (191/2112)		
Binge-eating		20.46% (433/2116)		
Excessive exercise		3.52% (74/2105)		
DSM-5 disordered eating		9.75% (204/2093)		
Any self-harm	8.97% (0.61)	8.75% (185/2115)		
Self-harm without suicidal intent		6.19% (131/2115)		
Self-harm with suicidal intent		1.68% (35/2081)		
Comorbid disordered eating and self-harm	5.53% (0.50)	5.36% (112/2089)		
Depressive symptoms during the pandemic	6.44 (0.12)	6.46 (0.13)	8.56 (0.29)	11.20 (0.58)
Anxiety symptoms during the pandemic	6.04 (0.11)	6.04 (0.12)	7.68 (0.25)	9.99 (0.50)
Mental wellbeing during the pandemic	44.26 (0.18)	44.24 (0.20)	41.78 (0.40)	38.79 (0.75)
Baseline depressive symptoms	6.90 (0.13)	6.85 (0.13)	9.16 (0.28)	12.88 (0.57)
Baseline anxiety symptoms	4.64 (0.10)	4.54 (0.11)	6.14 (0.23)	7.99 (0.44)
Baseline mental wellbeing	48.73 (0.19)	48.81 (0.20)	46.10 (0.40)	41.05 (0.75)

*Note:* Asterisks (“**”) indicate that estimates were reliant on cells containing five individuals or fewer. It is an ALSPAC stipulation that such data are not tabulated due to difficulties in maintaining participant anonymity for such rare characteristics. Descriptive information is only provided for variables included in the imputed datasets (i.e. those included in the main analysis).

**Table 2.** Association between disordered eating and self-harm exposures and pandemic mental health and wellbeing outcomes

	Unadjusted Model A		Adjusted Model B		Fully Adjusted Model C	
	
Exposure	B (95% CI)	p	B (95% CI)	p	B (95% CI)	p
***Outcome: Depressive symptoms***						
Disordered eating	2.98 (2.44, 3.53)	<.001	2.72 (2.17, 3.27)	<.001	1.37 (0.84, 1.90)	<.001
Self-harm	5.19 (4.31, 6.08)	<.001	4.95 (4.07, 5.83)	<.001	2.13 (1.24, 3.01)	<.001
Comorbid disordered eating and self-harm	6.15 (5.02, 7.29)	<.001	5.87 (4.74, 7.00)	<.001	2.52 (1.38, 3.66)	<.001
***Outcome: Anxiety symptoms***						
Disordered eating	2.43 (1.92, 2.95)	<.001	2.11 (1.59, 2.63)	<.001	1.24 (0.74, 1.74)	<.001
Self-harm	4.55 (3.74, 5.36)	<.001	4.26 (3.46, 5.05)	<.001	2.69 (1.87, 3.50)	<.001
Comorbid disordered eating and self-harm	5.38 (4.30, 6.47)	<.001	5.05 (3.98, 6.12)	<.001	3.08 (2.01, 4.15)	<.001
***Outcome: Mental wellbeing***						
Disordered eating	−3.49 (−4.30, −2.67)	<.001	−3.35 (−4.18, −2.51)	<.001	−1.82 (−2.59, −1.06)	<.001
Self−harm	−5.78 (−7.06, −4.50)	<.001	−5.62 (−6.90, −4.33)	<.001	−2.18 (−3.43, −0.93)	<.001
Comorbid disordered eating and self−harm	−7.81 (−9.43, −6.19)	<.001	−7.60 (−9.21, −6.00)	<.001	−3.64 (−5.19, −2.09)	<.001

Results using imputed data (n=2657). Model A = unadjusted; Model B = adjusted for sex, COVID1 questionnaire completion date and pre-pandemic socioeconomic status; Model C = adjusted for sex, COVID questionnaire completion date, pre-pandemic socioeconomic status and baseline mental health and wellbeing measures.

## List of abbreviations

ALSPAC: Avon Longitudinal Study of Parents and Children
GAD7: Generalised Anxiety Disorder 7-item questionnaire
MFQ: Mood and Feelings Questionnaire
WEMWBS: Warwick-Edinburgh Mental Well-Being Scale
