# Supplementary material for: Disordered eating and self-harm as risk factors for poorer mental health during the COVID-19 pandemic: A UK-based birth cohort study
Source: medRxiv. 2021 Nov 16:2021.04.30.21256377. Originally published 2021 May 4. Preprint. [Version 2] doi: 10.1101/2021.04.30.21256377 (PMC8109211; doi:10.1101/2021.04.30.21256377)

Supplementary Table 1. Comparison of responders and non-responders to the lifestyle change questions on the COVID1 questionnaire

**Variable**	**Description**	**n**	**Data on lifestyle changes**	**No data on lifestyle changes**	**χ^2**	**p**
Child gender	Male	2053	766 (28.83%)	1287 (48.11%)	209.32	<.001
	Female	3279	1891 (71.17%)	1388 (51.89%)		
Child ethnicity	White	4865	2455 (96.58%)	2410 (95.45%)	4.24	0.039
	Not white	202	87 (3.42%)	115 (4.55%)		
NEET at 23	In education, employment, or training	2967	1886 (94.96)	1081 (92.79%)	6.32	0.012
	NEET	184	100 (5.04%)	84 (7.21%)		
Maternal education	O level/GCSE and below	2,723	1289 (50.08%)	1434 (55.88%)	17.39	<.001
	A level and above (degree)	2,417	1285 (49.92%)	1132 (44.12%)		
Parity	First born	2,466	1267 (49.11%)	1199 (46.69%)	4.9	0.086
	Second born	1,794	895 (34.69%)	899 (35.01%)		
	Third or more born	888	418 (16.20%)	470 (18.30%)		
Home ownership	Don't own home	865	377 (14.58%	488 (18.91%)	17.37	<.001
	Own home	4,302	2209 (85.42%)	1093 (81.09%)		

Comparison amongst those sent the COVID1 questionnaire (n=5332). NEET = Not in education, employment, or training

Supplementary Table 2. Derivation of disordered eating and self-harm variables at age 24

**Questions**	**Answers**	**Coding**	**Final variable**
**Fasting**			
During the **past year**, how often did you fast (not eat for at least a day) to lose weight or avoid gaining weight?	0. Never<br>1. Less than once a month<br>2. 1-3 times a month<br>3. Once a week<br>4. More than once a week	Any fasting<br>0 = “no”<br>1-4 = “yes”<br>DSM fasting<br>0-2 = “no”<br>3-4 = “yes”	**Any fasting**
**Purging**			
During the **past year**, how often did you make yourself throw up to lose weight or avoid gaining weight?	0. Never<br>1. Less than once a month<br>2. 1-3 times a month<br>3. Once a week<br>4. More than once a week	Any self-induced vomiting<br>0 = “no”<br>1-4 = “yes”<br>DSM self-induced vomiting<br>0-2 = “no”<br>3-4 = “yes”	**Any purging**<br>Any self-induced vomiting<br>OR<br>Any laxative use<br>OR<br>Any other medication use
During the **past year**, how often did you take laxatives to lose weight or avoid gaining weight?	0. Never<br>1. Less than once a month<br>2. 1-3 times a month<br>3. Once a week<br>4. More than once a week	Any laxative use<br>0 = “no”<br>1-4 = “yes”<br>DSM laxative use<br>0-2 = “no”<br>3-4 = “yes”	
During the **past year**, how often did you take other tablets/pills/any other medications or substances to lose weight or avoid gaining weight?	0. Never<br>1. Less than once a month<br>2. 1-3 times a month<br>3. Once a week<br>4. More than once a week	Any other medication use<br>0 = “no”<br>1-4 = “yes”<br>DSM other medication use<br>0-2 = “no”<br>3-4 = “yes”	
**Excessive exercise**			
During the **past year**, how often did you exercise to **lose weight** or **avoid gaining weight**?	0. Never<br>1. Less than once a month<br>2. 1-3 times a month<br>3. 1-4 times a week<br>4. 5 or more times a week	Any exercise to lose weight<br>0 = “no”<br>1-4 = “yes”<br>DSM exercise to lose weight<br>0-2 = “no”<br>3-4 = “yes”	**Any excessive exercise**<br>Any exercise to lose weight<br>AND<br><br>Exercise interfered with life<br>OR<br>Exercise when sick/injured
Was it difficult for you to do your work or daily chores/routine because of the amount of time that you were exercising to lose weight or avoid gaining weight?	0. No<br>1. Yes, sometimes<br>2. Yes, frequently	Exercise interfered with life<br>0-1 = “no”<br>2 = “yes”	
Did you exercise to lose weight or avoid gaining weight even when you were sick or injured?	0. No<br>1. Yes, sometimes<br>2. Yes, frequently	Exercise when sick/injured<br>0-1 = “no”<br>2 = “yes”	
**Binge eating**			
Sometimes people will go on an ‘eating binge’, where they eat an amount of food that most people, like their friends	0. Never<br>1. Less than once a month<br>2. 1-3 times a month<br>3. Once a week	Any bingeing<br>0 = “no”<br>1-4 = “yes”<br><br>DSM bingeing	**Any binge eating**<br>Any bingeing<br>AND<br>Loss of control

or family, would consider to be very large in a short period of time. During the **past year**, how often did you go on an eating binge?	4. More than once a week	0-2 = "no"<br>3-4 = "yes"	
Do you ever feel like your eating is out of control, like you couldn't stop eating even if you wanted to?	0. No<br>1. Yes, sometimes<br>2. Yes, usually	Loss of control<br>0 = "no"<br>1-2 = "yes"	
**Any disordered eating**			
			**Any disordered eating**<br>Any fasting OR<br>Any purging OR<br>Any binge-eating OR<br>Any excessive exercise
**DSM-5 frequency disordered eating**			
		**DSM frequency purging**<br>DSM self-induced vomiting OR<br>DSM laxative use OR<br>DSM Other medication use<br><br>**DSM frequency binge-eating**<br>DSM bingeing AND<br>Loss of control<br><br>**DSM frequency excessive exercise**<br>DSM exercise to lose weight AND<br>Exercise interfered with life	**DSM-5 frequency disordered eating**<br>DSM frequency fasting OR<br>DSM frequency purging OR<br>DSM frequency binge-eating<br>OR<br>DSM frequency excessive exercise
**Self-harm for any reason**			
Have you **ever** hurt yourself on purpose in any way (e.g. by taking an overdose of pills or by cutting yourself)?	0. No<br>1. Yes	*Self-harm ever*<br>0 = "no"<br>1 = "yes"	**Self-harm in the last year**<br>*Self-harm past year*<br>(= 0 if self-harm ever = 0)
**If yes,** how many times have you done this in the last year?	0. None<br>1. Once<br>2. 2-5 times<br>3. 6-10 times<br>4. More than 10 times	*Self-harm past year*<br>0 = "no"<br>1-4 = "yes"	
**Non-suicidal self-injury**			
Have you **ever** hurt yourself on purpose (e.g. by taking an overdose of pills or by cutting yourself), without intending to kill yourself?	0. No<br>1. Yes	*NSSI ever*<br>0 = "no"<br>1 = "yes"	**NSSI in the last year**<br>*NSSI past year*<br>(= 0 if self-harm ever OR NSSI ever = 0)

If yes, when was the last time you hurt yourself on purpose, without intending to kill yourself?	1. In the last week<br>2. More than a week ago but in the last year<br>3. More than a year ago	*NSSI past year*<br>3 = "no"<br>1-2 = "yes"	
**Suicide attempt**			
On any of the occasions you have hurt yourself on purpose, have you **ever** seriously wanted to kill yourself?	0. No<br>1. Yes	*Attempt to kill ever*<br>0 = "no"<br>1 = "yes"	Suicide attempt ever<br>*Attempt to kill ever*<br>OR<br>*Reason: to die*
**In your lifetime,** do any of the following reasons help to explain why you hurt yourself?	a) I wanted to show how desperate I was feeling Y/N<br>b) I wanted to die<br>c) I wanted to punish myself<br>d) I wanted to frighten someone<br>e) I wanted to get relief from a terrible state of mind<br>f) Some other reason	*Reason: to die*<br>b 0 = "no"<br>b 1 = "yes"	
**If yes**, when was the last time you hurt yourself on purpose and you seriously wanted to kill yourself?	1. In the last week<br>2. More than a week ago but in the last year<br>3. More than a year ago	*Suicide attempt past year*<br>3 = "no"<br>1-2 = "yes"	**Suicide attempt in the last year**<br>*Suicide attempt past year*<br>(= 0 if suicide attempt ever = 0)

Supplementary Table 3. Question wording and variable coding for pre-pandemic measure of socioeconomic disadvantage (young person not in education, employment or training)

**Question wording**		**Possible responses**	**Final variable coding**
Are you currently			
	In full-time paid work (30 hours or more a week)	Yes (1)<br>No (0)	If all responses are No (0), then NEET variable is 1 (yes)<br><br>If any response is Yes (1), then NEET variable is 0 (No)
	In part-time paid work (less than 30 hours a week)	Yes (1)<br>No (0)	
	In irregular or occasional work	Yes (1)<br>No (0)	
	Doing a modern apprenticeship or other government supported training/work-experience scheme	Yes (1)<br>No (0)	
	In full-time education	Yes (1)<br>No (0)	
	Self-employed	Yes (1)<br>No (0)	

Supplementary Table 4. Question wording and variable coding for pandemic-related experiences

**Variable**	**Questionnaire Wording**	**Response Options**	**Final variable coding**
Living alone	Do you live with anybody?	No I live on my own<br>Yes, I live with at least 1 person	If "No I live on my own" then *living alone* = Yes (1)
Keyworker	Are you a keyworker, or has your work been classified as critical to the COVID-19 response?	Yes<br>No<br>Don't know	If "Yes" then *keyworker* = Yes (1), If "No" then *keyworker* = No (0)<br>If "Don't know" then *keyworker* = missing
Financial problems during the pandemic	Overall, how do you feel your current financial situation compares to how it was before the COVID-19 pandemic?	I'm much worse off<br>I'm a little worse off<br>I'm about the same<br>I'm a little better off<br>I'm much better off	If "I'm much worse off" or "I'm a little worse off" then *financial problems* = Yes (1)<br>Any other response then *financial problems* = No (0)
Furloughed during the pandemic	Which of these would you say best describes your current situation now?	Employed and working the same number of hours (as pre-lockdown)<br>Employed and working reduced number of hours<br>Employed and working more hours than before<br>Employed but on paid leave (including furlough)<br>Employed and on unpaid leave<br>Apprenticeship<br>In unpaid/voluntary work<br>Self-employed and currently working<br>Self-employed but not currently working<br>Unemployed<br>Permanently sick or disabled<br>Looking after home or family<br>In education at school/college/university	If "Employed but on paid leave (including furlough)" then *furlough* = Yes (1)<br>Any other response then *furlough* = No (0)

Supplementary Table 5. Amount of missing data on exposures, outcomes and confounders

**Variable**	**n present**	**n missing**	**% missing**
Primary exposures			
Any disordered eating	2096	561	21.11%
Any self-harm	2115	542	20.40%
Comorbid disordered eating and self-harm	2089	568	21.38%
Secondary exposures			
Fasting	2115	542	20.40%
Purging	2112	545	20.51%
Binge-eating	2116	541	20.36%
Excessive exercise	2105	552	20.78%
DSM-5 frequency disordered eating	2093	564	21.23%
Self-harm without suicidal intent	2115	542	20.40%
Self-harm with suicidal intent	2081	576	21.68%
Outcomes			
Depressive symptoms	1914	743	27.96%
Anxiety symptoms	1916	741	27.89%
Mental wellbeing	1922	735	27.66%
Confounders			
Gender	2657	0	0.00%
COVID1 questionnaire completion date	2657	0	0.00%
Pre-pandemic NEET	1986	671	25.25%
Pre-pandemic depressive symptoms	1998	659	24.80%
Pre-pandemic anxiety symptoms	1718	939	35.34%
Pre-pandemic mental wellbeing	2014	643	24.20%

NEET = Not in employment, education or training

Supplementary Table 6. Comparison of complete case and imputed analysis for main effects of disordered eating and self-harm on unstandardised pandemic mental health outcomes

			Complete case sample 1			Complete case sample 2			Imputed data		
			n	B (95% CI)	p	n	B (95% CI)	p	n	B (95% CI)	p
Outcome: depressive symptoms	DE	Model A	1341	3.26 (2.62, 3.90)	<.001	1585	3.09 (2.51, 3.68)	<.001	2657	2.98 (2.44, 3.53)	<.001
		Model B				1360	2.95 (2.31, 3.59)	<.001	2657	2.72 (2.17, 3.27)	<.001
		Model C				1341	1.48 (0.88, 2.09)	<.001	2657	1.37 (0.84, 1.90)	<.001
	SH	Model A	1350	5.37 (4.33, 6.41)	<.001	1594	5.29 (4.36, 6.22)	<.001	2657	5.19 (4.31, 6.08)	<.001
		Model B				1369	5.20 (4.18, 6.21)	<.001	2657	4.95 (4.07, 5.83)	<.001
		Model C				1350	2.34 (1.36, 3.33)	<.001	2657	2.13 (1.24, 3.01)	<.001
	DE+SH	Model A	1337	5.89 (4.59, 7.18)	<.001	1580	5.91 (4.72, 7.10)	<.001	2657	6.15 (5.02, 7.29)	<.001
		Model B				1356	5.61 (4.35, 6.87)	<.001	2657	5.87 (4.74, 7.00)	<.001
		Model C				1337	2.22 (1.01, 3.44)	<.001	2657	2.52 (1.38, 3.66)	<.001
Outcome: Anxiety symptoms	DE	Model A	1094	2.59 (1.97, 3.22)	<.001	1586	2.50 (1.97, 3.04)	<.001	2657	2.43 (1.92, 2.95)	<.001
		Model B				1359	2.32 (1.74, 2.90)	<.001	2657	2.11 (1.59, 2.63)	<.001
		Model C				1094	1.26 (0.66, 1.86)	<.001	2657	1.24 (0.74, 1.74)	<.001
	SH	Model A	1100	4.02 (3.03, 5.01)	<.001	1595	4.46 (3.62, 5.31)	<.001	2657	4.55 (3.74, 5.36)	<.001
		Model B				1368	4.05 (3.13, 4.96)	<.001	2657	4.26 (3.46, 5.05)	<.001
		Model C				1100	2.13 (1.19, 3.06)	<.001	2657	2.69 (1.87, 3.50)	<.001
	DE+SH	Model A	1090	4.52 (3.28, 5.75)	<.001	1581	5.09 (4.02, 6.17)	<.001	2657	5.38 (4.30, 6.47)	<.001
		Model B				1355	4.52 (3.39, 5.64)	<.001	2657	5.05 (3.98, 6.12)	<.001
		Model C				1090	2.27 (1.11, 3.43)	<.001	2657	3.08 (2.01, 4.15)	<.001
Outcome: mental wellbeing	DE	Model A	1343	-3.71 (-4.67, -2.75)	<.001	1592	-3.66 (-4.55, -2.77)	<.001	2657	-3.49 (-4.30, -2.67)	<.001
		Model B				1364	-3.72 (-4.69, -2.74)	<.001	2657	-3.35 (-4.18, -2.51)	<.001
		Model C				1343	-1.75 (-2.65, -0.85)	<.001	2657	-1.82 (-2.59, -1.06)	<.001
	SH	Model A	1351	-6.31 (-7.88, -4.73)	<.001	1601	-6.10 (-7.54, -4.67)	<.001	2657	-5.78 (-7.06, -4.50)	<.001
		Model B				1373	-6.14 (-7.70, -4.58)	<.001	2657	-5.62 (-6.90, -4.33)	<.001
		Model C				1351	-2.42 (-3.89, -0.94)	0.001	2657	-2.18 (-3.43, -0.93)	<.001
	DE+SH	Model A	1339	-8.09 (-10.02, -6.15)	<.001	1587	-8.10 (-9.91, -6.30)	<.001	2657	-7.81 (-9.43, -6.19)	<.001
		Model B				1360	-7.79 (-9.70, -5.87)	<.001	2657	-7.60 (-9.21, -6.00)	<.001
		Model C				1339	-3.45 (-5.26, -1.65)	<.001	2657	-3.64 (-5.19, -2.09)	<.001

Complete case sample 1 = Individuals with complete data on exposure, outcome and all confounders in the final model (n varies by model); Complete case sample 2 = all available observed data for each model (n varies by model); Imputed data = individuals with complete data on lifestyle changes with imputed data for exposures, outcomes and confounders (n=2657) (see manuscript Missing Data section).
DE = disordered eating; SH = Self-harm; DE+SH = comorbid disordered eating and self-harm.
Model A = unadjusted; Model B = adjusted for sex, COVID1 questionnaire completion date, pre-pandemic socioeconomic status; Model C = adjusted for sex, COVID1 questionnaire completion date, pre-pandemic socioeconomic status and pre-pandemic mental health and wellbeing symptoms.

Supplementary Table 7. Associations between secondary disordered eating and self-harm exposures and mental health outcomes during the pandemic

	**Unadjusted Model A**			**Adjusted Model B**			**Fully adjusted Model C**		
**Exposure**	**n**	**B (95% CI)**	**p**	**n**	**B (95% CI)**	**p**	**n**	**B (95% CI)**	**p**
Outcome: Depressive symptoms									
Fasting	1595	3.73 (2.83, 4.63)	<.001	1370	3.25 (2.27, 4.23)	<.001	1351	1.60 (0.70, 2.50)	<.001
Purging	1593	3.56 (2.60, 4.51)	<.001	1367	3.39 (2.35, 4.44)	<.001	1348	1.84 (0.89, 2.79)	<.001
Binge-eating	1596	2.68 (2.00, 3.36)	<.001	1370	2.57 (1.84, 3.30)	<.001	1351	1.25 (0.58, 1.92)	<.001
Excessive exercise	1592	2.17 (0.66, 3.69)	0.005	1368	1.95 (0.26, 3.64)	0.024	1349	0.75 (-0.75, 2.25)	0.324
DSM-5 frequency disordered eating	1583	3.75 (2.81, 4.69)	<.001	1360	3.58 (2.55, 4.61)	<.001	1341	1.93 (0.99, 2.87)	<.001
Self-harm without suicidal intent	1593	4.57 (3.48, 5.67)	<.001	1368	4.42 (3.24, 5.60)	<.001	1349	1.46 (0.35, 2.57)	0.01
Self-harm with suicidal intent	1569	8.00 (5.95, 10.05)	<.001	1349	9.22 (7.05, 11.39)	<.001	1330	4.88 (2.88, 6.88)	<.001
Outcome: Anxiety symptoms									
Fasting	1595	3.13 (2.31, 3.96)	<.001	1368	2.56 (1.67, 3.44)	<.001	1101	0.99 (0.08, 1.89)	0.032
Purging	1593	2.81 (1.93, 3.69)	<.001	1365	2.29 (1.34, 3.24)	<.001	1099	1.35 (0.42, 2.28)	0.005
Binge-eating	1596	2.08 (1.47, 2.70)	<.001	1368	1.97 (1.32, 2.63)	<.001	1100	1.19 (0.53, 1.86)	<.001
Excessive exercise	1593	1.81 (0.44, 3.19)	0.01	1367	1.65 (0.15, 3.14)	0.031	1099	1.79 (0.21, 3.36)	0.026
DSM-5 frequency disordered eating	1584	2.85 (1.99, 3.70)	<.001	1359	2.79 (1.87, 3.72)	<.001	1094	1.67 (0.71, 2.62)	0.001
Self-harm without suicidal intent	1594	3.47 (2.48, 4.46)	<.001	1367	3.21 (2.17, 4.26)	<.001	1100	1.70 (0.68, 2.73)	0.001
Self-harm with suicidal intent	1570	5.63 (3.73, 7.54)	<.001	1348	6.56 (4.57, 8.54)	<.001	1082	3.77 (1.73, 5.81)	<.001
Outcome: Mental wellbeing									
Fasting	1601	-4.76 (-6.11, -3.41)	<.001	1373	-4.22 (-5.69, -2.74)	<.001	1351	-1.76 (-3.10, -0.41)	0.011
Purging	1599	-3.81 (-5.27, -2.35)	<.001	1370	-3.74 (-5.33, -2.15)	<.001	1349	-1.49 (-2.93, -0.06)	0.041
Binge-eating	1602	-3.36 (-4.39, -2.34)	<.001	1373	-3.30 (-4.41, -2.20)	<.001	1351	-1.63 (-2.63, -0.63)	0.001
Excessive exercise	1599	-1.80 (-4.07, 0.47)	0.12	1372	-1.48 (-4.00, 1.04)	0.25	1350	-0.19 (-2.49, 2.12)	0.875
DSM-5 frequency disordered eating	1590	-4.14 (-5.57, -2.71)	<.001	1364	-3.97 (-5.54, -2.40)	<.001	1343	-1.82 (-3.25, -0.39)	0.013
Self-harm without suicidal intent	1600	-5.10 (-6.77, -3.43)	<.001	1372	-5.16 (-6.95, -3.37)	<.001	1350	-1.28 (-2.93, 0.37)	0.127
Self-harm with suicidal intent	1576	-10.28 (-13.45, -7.10)	<.001	1353	-12.20 (-15.57, -8.84)	<.001	1331	-6.46 (-9.58, -3.33)	<.001

Analyses conducted using all available observed data for each model. Model A = unadjusted; Model B = adjusted for sex, COVID1 questionnaire completion date, pre-pandemic socioeconomic status; Model C = adjusted for sex, COVID1 questionnaire completion date, pre-pandemic socioeconomic status and pre-pandemic mental health and wellbeing symptoms.

Supplementary Table 8. Associations between disordered eating frequency exposures and mental health outcomes during the pandemic

	**Unadjusted Model A**			**Adjusted Model B**			**Fully adjusted Model C**		
**Exposure**	**n**	**B (95% CI)**	**p**	**n**	**B (95% CI)**	**p**	**n**	**B (95% CI)**	**p**
Outcome: Depressive symptoms									
Disordered eating less than once a week	1585	2.54 (1.87, 3.20)	<.001	1360	2.43 (1.71, 3.15)	<.001	1341	1.17 (0.50, 1.83)	0.001
Disordered eating once a week or more		4.36 (3.42, 5.29)	<.001		4.25 (3.21, 5.28)	<.001		2.31 (1.35, 3.27)	<.001
Outcome: Anxiety symptoms									
Disordered eating less than once a week	1586	2.13 (1.52, 2.74)	<.001	1359	1.91 (1.27, 2.56)	<.001	1094	0.99 (0.33, 1.66)	0.003
Disordered eating once a week or more		3.35 (2.49, 4.21)	<.001		3.32 (2.38, 4.25)	<.001		1.97 (1.00, 2.95)	<.001
Outcome: Mental wellbeing									
Disordered eating less than once a week	1592	-3.13 (-4.15, -2.12)	<.001	1364	-3.25 (-4.35, -2.16)	<.001	1343	-1.54 (-2.54, -0.54)	0.002
Disordered eating once a week or more		-4.88 (-6.31, -3.45)	<.001		-4.87 (-6.45, -3.29)	<.001		-2.31 (-3.77, -0.85)	0.002

Analyses conducted using all available observed data for each model. Reference category is no disordered eating (0).

Model A = unadjusted; Model B = adjusted for sex, COVID1 questionnaire completion date, pre-pandemic socioeconomic status; Model C = adjusted for sex, COVID1 questionnaire completion date, pre-pandemic socioeconomic status and pre-pandemic mental health and wellbeing symptoms.

Supplementary Table 9. Lifestyle changes during lockdown (n=2657)

**Since lockdown the participant has changed the amount:**	**Decrease**		**Stayed the same**		**Increase**	
	n	%	n	%	n	%
they *sleep*	628	23.64	1,152	43.36	877	33.01
of physical activity/*exercise*	1,151	43.32	697	26.23	809	30.45
of *alcohol* drunk	397	14.94	1,362	51.26	898	33.80
visiting *green space*	1,207	45.43	692	26.04	758	28.53
they *eat*	241	9.07	1,319	49.64	1,097	41.29
of time spent *talking* to family/friends outside their home	982	36.96	524	19.72	1,151	43.32
of time spent doing *hobbies*/things they enjoy	478	17.99	891	33.53	1,288	48.48
of practising *relaxation*/mindfulness/meditation	136	5.12	2,021	76.06	500	18.82

Supplementary Table 10. Associations between disordered eating/self-harm exposures (age 25 years, YPD questionnaire) and lifestyle change moderators (COVID1 questionnaire)

**Exposure**	**Lifestyle change moderator**	**Decreased (ref = stayed same)**		**Increased (ref = stayed same)**		
		**RRR**	**95% CI**	**RRR**	**95% CI**	**p**
Disordered eating	Sleep	2.055	[1.630, 2.589]	1.568	[1.267, 1.939]	< 0.0001
Disordered eating	Exercise	1.214	[0.974, 1.511]	1.039	[0.818, 1.320]	0.1592
Disordered eating	Alcohol	0.971	[0.744, 1.267]	1.292	[1.062, 1.573]	0.0217
Disordered eating	Green space	1.333	[1.062, 1.673]	1.098	[0.850, 1.419]	0.0294
Disordered eating	Eating	1.732	[1.251, 2.398]	1.881	[1.547, 2.286]	< 0.0001
Disordered eating	Talking to friends/family	1.170	[0.903, 1.516]	1.117	[0.871, 1.433]	0.4783
Disordered eating	Hobbies	1.080	[0.828, 1.408]	1.111	[0.906, 1.363]	0.6001
Disordered eating	Relaxation	1.335	[0.900, 1.979]	1.123	[0.887, 1.422]	0.2642
Self-harm	Sleep	1.658	[1.165, 2.361]	1.056	[0.741, 1.505]	0.0109
Self-harm	Exercise	1.138	[0.805, 1.610]	0.711	[0.473, 1.068]	0.0400
Self-harm	Alcohol	0.884	[0.569, 1.372]	0.966	[0.703, 1.328]	0.8574
Self-harm	Green space	1.100	[0.777, 1.556]	0.741	[0.487, 1.129]	0.1109
Self-harm	Eating	1.719	[1.059, 2.791]	1.230	[0.895, 1.689]	0.0759
Self-harm	Talking to friends/family	1.362	[0.874, 2.122]	1.325	[0.861, 2.040]	0.3536
Self-harm	Hobbies	1.189	[0.793, 1.783]	0.912	[0.660, 1.261]	0.3984
Self-harm	Relaxation	2.489	[1.469, 4.219]	1.098	[0.753, 1.602]	0.0026

Results using imputed data (n=2657).

Supplementary Table 11. Unadjusted associations between lifestyle change moderators (COVID1 questionnaire) and pandemic mental health and wellbeing outcomes (COVID2 questionnaire)

**Lifestyle change**	**Mental health outcome**	**Decreased (ref = stayed same)**		**Increased (ref = stayed same)**		
		**Diff (SE(Diff))**	**95% CI**	**Diff (SE(Diff))**	**95% CI**	**p**
Sleep	Depressive symptoms	0.564 (0.052)	(0.462, 0.666)	0.224 (0.047)	(0.132, 0.317)	< 0.0001
Exercise	Depressive symptoms	0.234 (0.051)	(0.134, 0.333)	0.015 (0.056)	(-0.094, 0.124)	< 0.0001
Alcohol	Depressive symptoms	-0.007 (0.060)	(-0.126, 0.111)	0.101 (0.047)	(0.009, 0.194)	0.0655
Green space	Depressive symptoms	0.235 (0.050)	(0.136, 0.333)	0.065 (0.056)	(-0.044, 0.174)	< 0.0001
Eating	Depressive symptoms	0.431 (0.075)	(0.283, 0.579)	0.231 (0.043)	(0.146, 0.317)	< 0.0001
Talking to friends/family	Depressive symptoms	0.114 (0.058)	(0.001, 0.226)	0.064 (0.057)	(-0.047, 0.175)	0.1426
Hobbies	Depressive symptoms	0.165 (0.061)	(0.045, 0.285)	-0.030 (0.047)	(-0.123, 0.063)	0.0033
Relaxation	Depressive symptoms	0.500 (0.098)	(0.307, 0.693)	0.134 (0.055)	(0.026, 0.241)	< 0.0001
Sleep	Anxiety symptoms	0.662 (0.052)	(0.560, 0.764)	0.252 (0.046)	(0.161, 0.342)	< 0.0001
Exercise	Anxiety symptoms	0.154 (0.051)	(0.053, 0.255)	0.053 (0.056)	(-0.056, 0.162)	0.0074
Alcohol	Anxiety symptoms	0.011 (0.060)	(-0.107, 0.129)	0.045 (0.047)	(-0.046, 0.137)	0.6121
Green space	Anxiety symptoms	0.199 (0.051)	(0.099, 0.300)	0.080 (0.056)	(-0.029, 0.190)	0.0003
Eating	Anxiety symptoms	0.513 (0.074)	(0.368, 0.659)	0.232 (0.044)	(0.146, 0.318)	< 0.0001
Talking to friends/family	Anxiety symptoms	0.128 (0.058)	(0.014, 0.242)	0.118 (0.057)	(0.006, 0.229)	0.0641
Hobbies	Anxiety symptoms	0.133 (0.061)	(0.013, 0.252)	-0.025 (0.046)	(-0.116, 0.066)	0.0216
Relaxation	Anxiety symptoms	0.548 (0.095)	(0.362, 0.734)	0.236 (0.054)	(0.130, 0.341)	< 0.0001
Sleep	Mental wellbeing	-0.540 (0.053)	(-0.644, -0.437)	-0.095 (0.048)	(-0.189, -0.002)	< 0.0001
Exercise	Mental wellbeing	-0.192 (0.051)	(-0.292, -0.093)	0.061 (0.056)	(-0.048, 0.170)	< 0.0001
Alcohol	Mental wellbeing	-0.004 (0.061)	(-0.123, 0.116)	-0.086 (0.047)	(-0.177, 0.005)	0.1568
Green space	Mental wellbeing	-0.208 (0.052)	(-0.310, -0.107)	-0.028 (0.057)	(-0.140, 0.083)	< 0.0001
Eating	Mental wellbeing	-0.264 (0.074)	(-0.410, -0.118)	-0.222 (0.045)	(-0.309, -0.134)	< 0.0001
Talking to friends/family	Mental wellbeing	-0.049 (0.058)	(-0.163, 0.065)	0.043 (0.057)	(-0.069, 0.156)	0.1441
Hobbies	Mental wellbeing	-0.083 (0.061)	(-0.203, 0.036)	0.191 (0.048)	(0.097, 0.285)	< 0.0001
Relaxation	Mental wellbeing	-0.469 (0.097)	(-0.658, -0.279)	0.099 (0.055)	(-0.009, 0.206)	< 0.0001

Results using imputed data (n=2657).

Supplementary Figure 1. Participant attrition

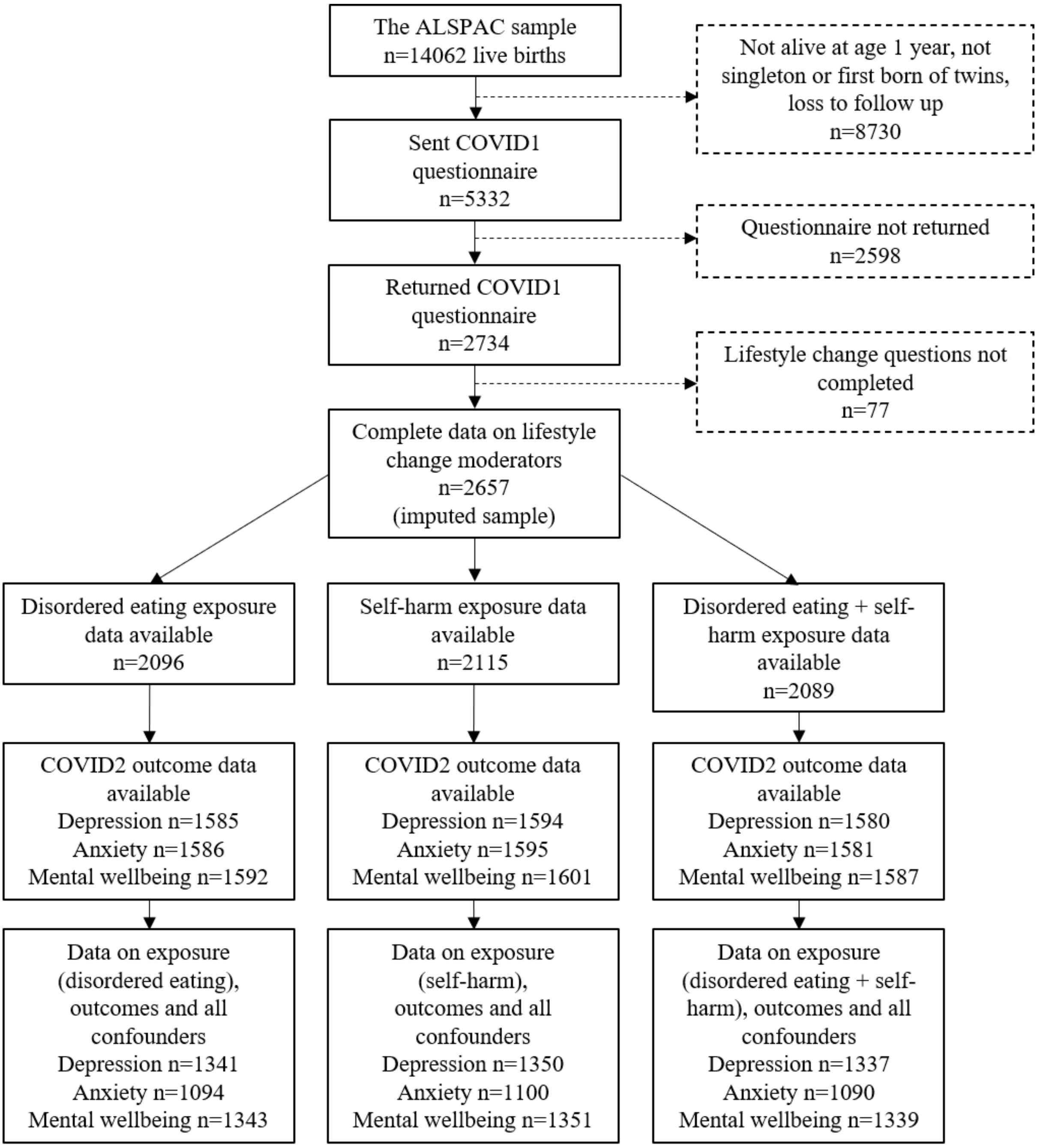

Supplement: Supplement 1 [file media-1.pdf]
